# Soft-core processor integration based on different instruction set architectures and field programmable gate array custom datapath implementation

**DOI:** 10.7717/peerj-cs.1300

**Published:** 2023-04-18

**Authors:** Ionel Zagan, Vasile Gheorghiţă Găitan

**Affiliations:** 1 Faculty of Electrical Engineering and Computer Science, Stefan cel Mare University, Suceava, Romania; 2 Integrated Center for Research, Development and Innovation in Advanced Materials, Nanotechnologies, and Distributed Systems for Fabrication and Control (MANSiD), Stefan cel Mare University, Suceava, Romania

**Keywords:** Field-programmable gate arrays (FPGAs), MIPS32, MicroBlaze, ARM, Asynchronous event handling

## Abstract

One of the fundamental requirements of a real-time system (RTS) is the need to guarantee re-al-time determinism for critical tasks. Task execution rates, operating system (OS) overhead, and task context switching times are just a few of the parameters that can cause jitter and missed deadlines in RTS with soft schedulers. Control systems that are susceptible to jitter can be used in the control of HARD RTS as long as the cumulative value of periodicity deviation and worst-case response time is less than the response time required by that application. This artcle presents field-programmable gate array (FPGA) soft-core processors integration based on different instruction set architectures (ISA), custom central processing unit (CPU) datapath, dedicated hardware thread context, and hardware real-time operating system (RTOS) implementations. Based on existing work problems, one parameter that can negatively influence the performance of an RTS is the additional costs due to the operating system. The scheduling and thread context switching operations can significantly degrade the programming limit for RTS, where the task switching frequency is high. In parallel with the improvement of software scheduling algorithms, their implementation in hardware has been proposed and validated to relieve the processor of scheduling overhead and reduce RTOS-specific overhead.

## Introduction

As demonstrated in practice, Moore’s law validated the theory of continuous scaling and miniaturization of transistors in an integrated circuit (IC). This theory, along with the concept of abstraction, has guided the semiconductor industry to the present day leading to the emergence of System on Chip (SoC), hybrid scheduling (Ghavidel, Sedaghat & Naghibzadeh, 2020; Aurora Dugo et al., 2022), heterogeneous multicore processors (Pei, Kim & Gaudiot, 2016a; Krishnakumar et al., 2020), and hardware microkernels (Dantas, De Azevedo & Gimenez, 2019a), today’s computing systems (Bae, 2021) and Internet of Things (IoT) concepts. With technological development, designers of central processing units have developed modern IC in various forms such as FPGAs (Li et al., 2022), complex programmable logic devices (CPLDs), or application-specific integrated circuits (ASICs), which are faster and smaller, consume less power and, last but not least, are cheaper. In current practical research, they continue to improve the performance of processors, ISAs, and RTOSs by multiplying thread contexts, integrating scheduling algorithms into the hardware, and minimizing the response time for the entire RTS (Dantas, De Azevedo & Gimenez, 2019b; Pei, Kim & Gaudiot, 2016b).

RTS holds a primordial place in today’s society because most systems used to facilitate automation processes are controlled by microprocessors. The specific role of RTS is to provide predictable and deterministic control of a process. RTS are those systems that provide a correct response within a predetermined time frame. The response speed is not a specific feature of RTS, it is rather an abstract term in the automation process. Events jitter, however, is a characteristic RTS specific that is separate from the one mentioned above. For that reason, and perhaps because of a blurred picture of the subject, some engineers have considered that RTS research is not a future-oriented field because the continuous increase in processor speed will produce equipment fast enough to meet the requirements of the most demanding applications. In reality, task execution speed does not imply a guaranteed scheduling scheme for all task sets in the system. Specialized processors exploit the massively parallel in-memory processing capability of DRAM to execute non-deterministic finite automata, improving performance over traditional architectures (Mittal, 2019).

The emergence of reduced instruction set computer (RISC) architecture had a dramatic start in terms of the historical trend in processor architecture. Even though this architecture has been defined and designed in a variety of ways by different groups, its key elements are as follows:

• A large number of general purpose registers (GPR) that partially compensate for the lack of memory instructions and the use of compiler technology to optimize the use of these registers;
• A simple and limited instruction set, about 35 instructions of fixed length (32 bits) for MIPS32, and a small number of addressing modes;
• A special focus on optimizing the pipeline, enabling the execution of instructions in minimum time.

A first feature of the RISC architecture is a machine instruction per machine cycle. A machine cycle is defined as the time required to fetch two operands from GPR, perform an arithmetic or logical operation and store the result in a register. Therefore, RISC processor instructions should not be more complicated and their execution must be at least as fast compared to complex instruction set architecture (CISC) processor micro-instructions. However, new RISC architectures are proposed to improve some aspects of ISA. For example, in RISC-V ISA there are four types of instruction formats R, I, S and U (Gruin et al., 2021a), then there is a variant of the S and U types, which are SB and UJ. In order to minimize the combinational delays in the decoding stage, the RISC-V instruction set architecture places the most important fields in the same place in each instruction. Thus, for the immediate field, the bits are shuffled in the instruction format. Register-to-register operation is another feature of the RISC architecture, as a simpler control unit and a simplified and optimized instruction set was required for the use of registers containing frequently accessed operations.

The CISC architecture also provides such instructions, additionally including mixed memory to memory and register/memory operations. On the other hand, almost all RISC instructions use simple register-level addressing (Patterson & Hennessy, 2011). Some additional addressing modes such as displacement and program counter (PC) relative can be included, other more complex modes can be synthesized in software. Another feature of the RISC architecture is the simple instruction format, favouring many practical implementations such as PIC32 or advanced RISC machine (ARM). Thus, the fixed and aligned instruction length, the fixed location of the OpCode field as well as a simplified control unit allow opcode decoding and operand register access to be performed simultaneously. Comparing the advantages of RISC and CISC architectures, it can be stated that RISC processors can improve their performance by implementing CISC features, and designs based on CISC architectures such as the Pentium II can benefit from certain RISC features.

Due to the complexity and high number of automation applications and response times imposed in RTS, existing problems such as “robot axes do not move smoothly”, “robot control accuracy is diminished” and “network performance is insufficient” may exist when software RTOSs are integrated. The challenges in real-time embedded systems are very rigorous, and some RTS cannot use RTOS, because in some cases, the RTOS overhead is too high or the system does not reach the required performance. These architectural and implementation aspects result in the following drawbacks: it is difficult to add or modify software; large-scale software design is cumbersome; very expensive to modularize and upgrade software.

The research gap could be filled by HW implementation of certain RTOS functions resulting in the concept of HW-RTOS. However, despite the implementation in the FPGA of necessary resources, HW-RTOS offers a high level of real-time performance, supported by fast execution of the API and guaranteeing fast response to the interrupt, reduced RTOS overhead and footprint, tick offloading, HW ISR (hardware interrupt service routine) and significantly lower CPU resource usage.

The research motivation behind this research project is the minimization of task context switching time and the implementation of a predictable event-based hardware scheduler. The proposed HW-RTOS validates and provides excellent real-time performance with low hardware and software overhead compared to conventional software RTOS implementations used in the industry. This architectural aspect allows specifying a worst-case execution time, which guarantees the design of predictable real-time systems.

The main contributions are the following:

• The proposal of a solution to minimize the task context switching time (based on a proposed patent for the concept of CPU resource multiplication);
• The implementation of a flexible and versatile scheme for handling time events, mutex, message and interrupt type events attached to a task, *i.e.,* these events can be prioritized at the thread level;
• The proposal of an algorithm for handling interrupt events implemented in nHSE hardware scheduler.

This article begins with an introduction in Section 1, and Section 2 presents the authors’ proposed articles in the literature. Section 3 presents the experimental resources and Section 4 describes soft-core FPGA integration processor based on custom datapath implementations. Finally, Section 5 concludes this article presenting the improvements brought by this research.

## Related Work

In RENESAS (2018), elements that introduce overcontrol relative to the runtime of RTOS mechanisms are identified and measured. An RTOS is often used in embedded systems for several reasons. These include the fact that it is easier to create a multi-task environment using RTOS, and the use of semaphores and events specific to inter-task communication simplifies the implementation of inter-task synchronization and communication. This results in easier modularization and reuse of software, thus improving software development productivity as well as improved reliability of the designed system.

This article addresses specific scheduling methods within two-stage real-time systems (2S-RTS) that schedule and execute aperiodic tasks considering firm and soft deadlines (Leng et al., 2020). The authors propose and validate a new sharing-based heuristic scheduling algorithm called HS-2S-RTS. The tested scheduling algorithm can achieve efficient online scheduling and ensures all the strict constraints on the imposed deadlines. The implementation of this project also maximizes the minimum CPU share allocated to soft tasks, the schedulability of both firm and soft tasks can be improved in the context of RTS.

In article (Nordström et al., 2005), the authors use TRON project research and implement a processor using a hardware component called real-time unit (RTU). The RTU component is realized in the hardware description language VHDL and is composed of a scheduler that provides communication between processes. It has been developed as an Intellectual Property (IP) component. The use of the component is achieved through a set of registers located inside the main RAM and a software kernel, about 2 Kb in size, which allows the scheduler to interact with the hardware kernel. Experimental results revealed that the shortest response time of a system call, is much lower in the case of the RTU component, only when the hardware part of the RTU is compatible with the software part of the µC/OS-II operating system.

Recent real-time systems need an increase in processing power, leading to the adoption of single and multi-core processors. However, single-core processors are proposed to incorporate acceleration mechanisms that combine out-of-order executions, complex pipelines, caches and in-branch speculation. In Gruin et al. (2021b) MINOTAuR, an open source time-predictable RISC-V core based on the Ariane core is presented. The authors first modify Ariane to make it time predictable, following the approach used for processor design.

If the scheduling algorithm is implemented in hardware, the scheduling process is accelerated. Experimental results, presented in the article (Gupta et al., 2010), show that performance tests run for the three types of implementations validate the hardware scheduler is about 5 times more efficient than software implementations and three times faster when it comes to task scheduling.

**Figure 1:**
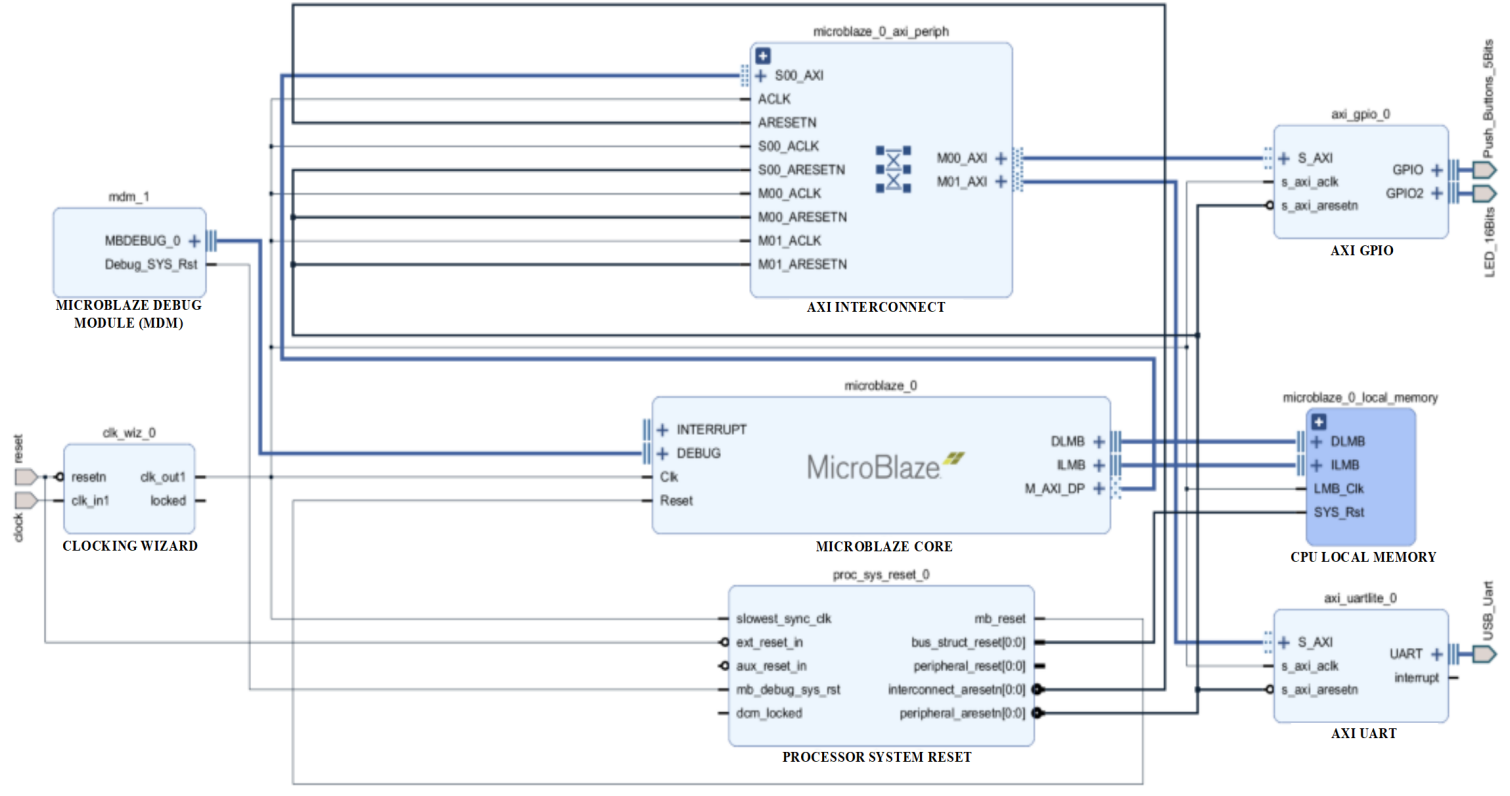
Block design implementation based on MicroBlaze soft-core and Artix-7 FPG.

Based on the idea of abstraction within the computer architecture and at the same time the rapid development of FPGA circuits, MicroBlaze soft-core was proposed (AMD, 2017). The MicroBlaze processor in an Arty SoC configuration has an operating frequency of 100 MHz, although it can operate at over 200 MHz. Once the soft SoC configuration for Arty is vasidated and designed, embedded system designers can write and debug programs for this RISC soft-core proposed by Xilinx. The design methodology involves exporting the SoC design from the Vivado IPI to the Xilinx Software Development Kit (XSDK), which is an integrated development environment for designing programs in C using MicroBlaze (Fig. 1). After transferring the IPI to the XSDK, it is automatically configured to include libraries corresponding to the included peripheral blocks. So FPGA-based design and Arty programming is very similar to using other SoC platforms or microcontrollers. In this architectural context programs are written in C, later loaded into the Xilinx FPGA *via* USB and then optionally debugged in hardware with appropriate tools.

The article (Chen et al., 2019) proposes and validates a particular method of reducing the communication frequency between CPU and FPGA for reconfigurable hybrid hardware. Thus, the processing speed of the tested design is higher by simplifying the communication between CPU and FPGA since it divides the software into master hardware execution threads and slave execution threads. These execution threads have the ability to run independently on the FPGA, the CPU only needs to take the output from the reconfigurable circuit. This reduces unnecessary communication time as the control logic part of the CPU is implemented in the FPGA. However the main execution thread in the FPGA is responsible for the hardware processing control logic.

Vector processors promise improved energy efficiency for data-parallel workloads (Platzer & Puschner, 2021). They also have the potential to reduce the performance gap between platforms suitable for time-critical applications and mainstream processors. Current trends for improving energy efficiency and the emergence of massive parallel data workloads have prompted massive research for architectures that may be more amenable to WCET analysis. Vector processors are also very important for real-time applications with parallel data processing. Some examples of applications in this category would be advanced driver assistance systems and autonomous vehicles.

In article (Pujari, Wild & Herkersdorf, 2015), the authors present a new approach for a Network On Chip (NOC) architecture, where each processor core has a task control unit, called a TCU (Thread control unit). This unit schedules tasks based on sensor information and according to the importance of each task. Each TCU calculates the cost for each task and selects the smallest one. The authors proposed, for the practical part, an architecture with two MPSoC cores, which are interconnected by a NOC link, using the Virtex6 FPGA development board. Each MPSoC core contains four Leon3 RISC processors and a TCU.

In article (Nacul, Regazzoni şi & Lajolo, 2007), a hardware-implemented RTOS (HW-RTOS) integrating an OS based on dual-core processor Symmetric MultiProcessor (SMP) architecture. Task communication is specified at the software interface level, and the HW-RTOS handles the application communication and task scheduling requirements. HW-RTOS is able to use the task migration provided by the SMP architecture much more efficiently than a traditional RTOS system. Dual-core processor architecture contains two processors with cache memory, data memory and a common bus. The HW-RTOS is composed of two independent scheduling modules for each processor. Each module communicates with the controlled ARM processor through a dedicated port. This architecture uses a hardware scheduler only to schedule tasks, and context switching is done in software. Only communication between tasks and access to shared memory is done in hardware. The performance of the architecture has been measured using two applications: an application that filters a graph representing typical operations performed by the kernel of a multimedia application, and an application using a kernel that processes packet-based communication.

The proposed design described in Coluccio et al. (2022) replaces the data memory with a circuit that is capable of storing data and performing calculations in memory, respectively. In this context, the authors propose a RISC-V framework that supports logic-in-memory operations. The results presented by the authors demonstrate an improvement in algorithm execution speed and also a reduction in energy consumption. Note that the main advantage of this framework is the ability to compare the performance of different logic-in-memory solutions at code execution. Since the framework is based on a standard memory interface, different logic-in-memory architectures, based on both CMOS and emerging technologies, can practically be placed inside the microprocessor. In this article (Coluccio et al., 2022), the efficiency of the framework is verified using a CMOS volatile memory and a memory based on a new emerging technology, race circuit logic.

Recent SoC implementations are often considered for analysis, they are evaluated for processing performance, FPGA area and resource utilization, power consumption and efficiency. In Doerflinger et al. (2021) the authors compare leading open-source RISC-V application class designs, running identical benchmarks on design platforms but with defined configuration settings. However, the experimental data obtained helps to make the right choice of designers for future projects with increasingly different processing needs. The authors present results for the Xilinx Virtex UltraScale+ family and GlobalFoundries 22FDX ASIC technology, so it can be stated that the large variations in results highlight the importance of processor selection for SoC implementations. The tests demonstrate that the ranking order depends on the selected technology, which can be FPGA or ASIC, and the primary requirements such as efficiency, cost or performance. Clearly, there is no generally optimal implementation for choosing a processor with a particular hardware design platform for that architecture.

The commercial RTOS core µC/OS-II (Labrosse, 2002) was implemented in C language with small pieces of code written in assembly language. The Real-Time Unit (RTU) component was used to replace the task scheduling, semaphore management, and specialized registers in the µC/OS-II operating system. The experimental results, presented in this article, revealed that the shortest response time of a system call, is much lower in the case of the RTU component, only when the hardware part of the RTU is compatible with the software part in the µC/OS-II operating system. For better compatibility, the RTU component requires modifying the bus interface and expanding the size of the data transferred to 32 bits. This will increase the number of processor cycles to set up a timeout and will shorten the response time of a system function group time-out call to the RTU. Another enhancement to the RTU would be to add the ability for tasks to support dynamic priority for better compatibility with the µC/OS-II operating system.

## Experimental Resources

In the research related to this article, the main resources used are the Virtex-7 development kit, Vivado DS, Verilog HDL, oscilloscope, personal computer, Vivado simulator, and MIPS32 ISA. The major advantages brought by this development platform based on Virtex-7 programmable logic technology are guaranteed high performance relative to power consumption, integration using 28 nm technology, Digital Signal Processing (DSP) performance, and I/O bandwidth. The XC7VX485T-2ffg1761C FPGA circuit features 485760 Logic Cells, maximum 8175 Kb Distributed RAM, 1030 Block RAM/FIFO w/ECC (36 Kb each), 2800 DSP Slices, one Analog Mixed Signal/XADC module, as well as other important resources.

The XC7VX485T FPGA is composed of three main elements: Look-Up Tables (LUTs), Flip-Flops (FFs), and routing channels. These representative elements in programmable logic technology are connected together to form a flexible and high-performance device. A LUT is a table that determines how the output is affected by any of the signals present at the inputs. Thus, a LUT consists of a RAM block that is indexed by its entries. The output of a LUT is represented by the value in the RAM location indexed by the inputs. In the context of combinational logic, this is represented by the table of truth, which effectively defines how the implemented circuit behaves.

Microprocessor without Interlocked Pipeline Stage (MIPS) provides a system of coprocessors (COP) to extend the core functionality of the processor. COP2 may be available to the user. MIPS Application Specific Extensions (ASE) and User Defined Instructions (UDI) are two other important aspects. Thus, the MIPS32 and MIPS64 architectures provide robust support for user application-specific extensions. As optional extensions to the base architecture, they do not supplement each implementation of the architecture with instructions or capabilities that are only required for a particular implementation. The MIPS32 and MIPS64 architectures allow specific UDI for each implementation, which is additional support for ASE. Thus, the Special 2 and COP2 fields are reserved for the capability defined by each implementation (Ciobanu, 2018). Based on MIPS32 ISA (Anonymous, 2011), the new instructions specific to the nHSE (hardware scheduler engine for n threads) have been implemented, a more extensive presentation of which can be found in the HW_nMPRA_RTOS (a unified acronym for nMPRA, nHSE, and RTOS API) processor specifications (Gaitan, Gaitan & Ungurean, 2015). Table 1 presents the notations and details in the datapath used for the proposed MIPS32 ISA based project. Resources in the datapath have been multiplied n times (HW_thread_i), so a hardware instance for the thread i is denoted by instPi. The preemptive scheduler switches between instPi threads executing in its own HW_thread_i, providing a context switching time of up to two processor cycles. External interrupt, time, deadline, mutext, and message synchronization events are dynamically attached to instPi, inheriting its priority. Using COP2 instructions implemented for nHSE scheduler, the interrupt event system, including their individual management, can be managed with minimal jitter. COP2 dedicated instructions are decoded independently beside program instructions, based on the instruction fetch/decode pipeline register information.

**Table 1:** HW_nMPRA_RTOS notations, nHSE registers implemented in COP2 and datapath acronyms.

Notation	Number of instances	Description
nMPRA	1	Multi pipeline register architecture where n is the degree of (MIPS32/RISC-V/ARM Cortex-Mx) datapath resource multiplication
nHSE	1	Hardware scheduler engine for n threads
HW_nMPRA_RTOS	1	Unified acronym for nMPRA, nHSE and RTOS API
HW_thread_i	4 ÷32	Private resources of threads (incorporates the PC, register file, pipeline registers and control&status registers)
instPi (sCPUi)	4 ÷32	Hardware instances of a thread executed on HW_thread_i type resource
crTRi	4 ÷32	Control task register has the role of validating or inhibiting one of the following events at the level of each instPi: lr_enTi (time event), lr_enWDi (watchdog timer), lr_enD1i (deadline 1), lr_enD2i (deadline 1), lr_enInti (interrupt type event), lr_enMutexi (mutex), lr_enSMi (signal and message) and lr_run_instPi
crEVi	4 ÷32	Control events register has the role of indicating the occurrence of an event validated by crTRi at the level of each instPi
crEPRi	4 ÷32	Control events priority register has the role of prioritizing events at the level of each instPi
ExtIntEv	4	External interrupt signals connected to nHSE module
grINT_IDi	4 ÷32	Task ID for interrupt attach register

## Custom Soft-Core Processor FPGA Development and Integration

The MIPS instruction set architecture has evolved from the original MIPS I™ ISA to the current MIPS32^®^, MIPS64^®^, and micro-MIPS™ versions. In the MIPS III™ version, integers and 64-bit addresses were introduced and in the MIPS IV™ and MIPS V™ ISAs, improvements were made to floating point operations as well as the instruction set to increase the efficiency of the generated code and data flow. Thus, MIPS implementations have had significant success in the embedded systems domain, with a focus on applications that require a focus on implementation cost, performance, and power consumption. However, many of the original MIPS implementations were targeted at desktop applications such as servers and workstations.

The MIPS32 and MIPS64 architectures are intended to address applications with a higher performance requirement for the MIPS-specific instruction set. They offer a high cost/performance ratio compared to other microprocessor implementations based on traditional architectures. The MIPS32 architecture is not tied to a specific hardware implementation, so CPU architects can design their own hardware concepts. These advantages are due to improvements in several research areas such as processor organization, system-level architectures, very large-scale integration (VLSI) technology, OSs, and compiler design. The MIPS architecture defines four coprocessors, namely COP0, COP1, COP2, and COP3. Coprocessor 0 is integrated into the CPU being called System Control Coprocessor and is defined to support both a virtual memory system and exception handling. COP0′s role includes translating virtual addresses into physical addresses, cache subsystem control, exception management, and handling of switches between core, supervisor, and user states as well as providing a diagnostic model and error recovery. COP1 is reserved for FPU while COP2 is available for particular implementations. Starting with the Release 1 implementation belonging to MIPS64 and in all Release 2 implementations of the MIPS architecture, COP3 is intended for the FPU.

### Proposed soft-core processor datapath multiplication

**Figure 2:**
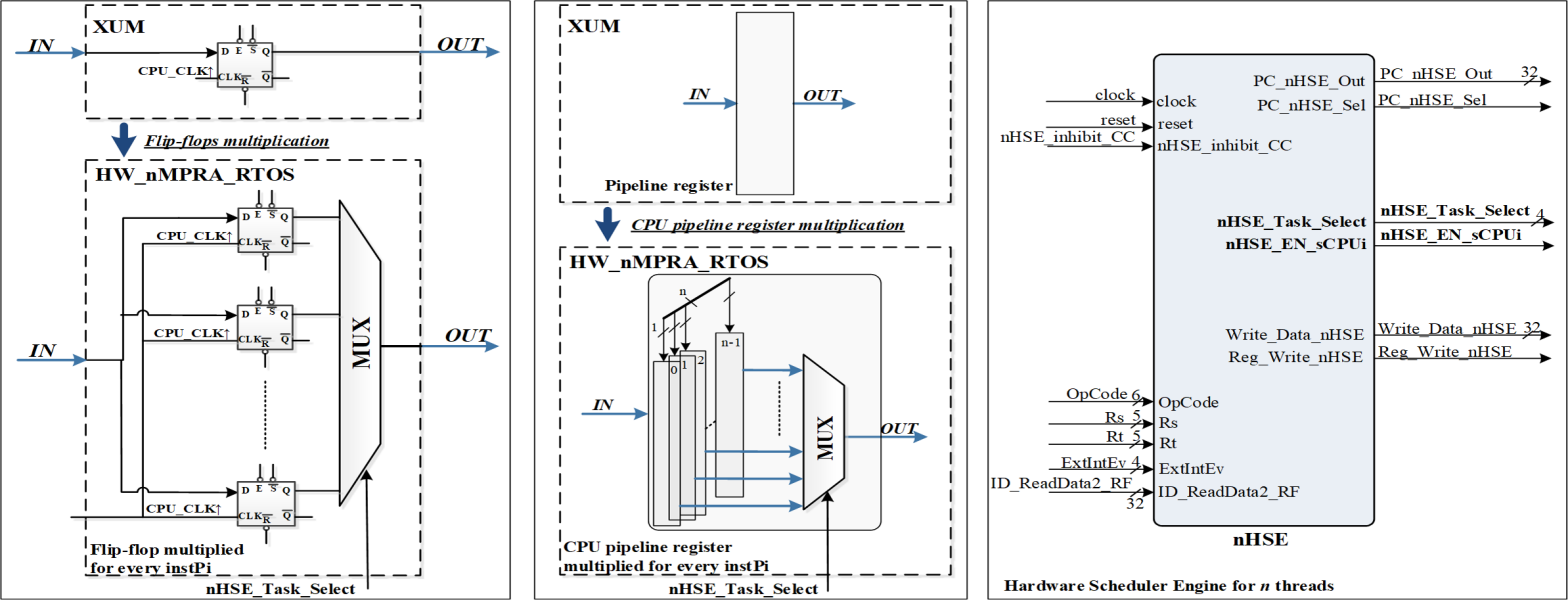
Resource multiplication within the HW_nMPRA_RTOS architecture relative to the XUM processor.

The HW_nMPRA_RTOS (nMPRA (multi pipeline register architecture, where n is the degree of multiplication) + nHSE) project datapath presented in this article used the MIPS32 Release 1 ISA (Ayers, 2020; Meakin, 2010). The HW_nMPRA_RTOS implementation validated in this article is based on the XUM design described in Ayers (2020), which is a five-stage pipeline MIPS32 processor. Figure 2 shows the multiplication of resources at the level of each storage element (flip-flop) in the datapath, *i.e.,* at the level of an n-fold multiplied pipeline register.

 Based on the status signals, the control signals generated by the nHSE module are nHSE_Task_Select, nHSE_EN_sCPUi, PC_nHSE_Sel, and Reg_Write_nHSE. These signals control the operation of datapath multiplexing, event trap cell selection, and writing to COP2 registers.

Also shown in Fig. 2 is the nHSE module controlling the dedicated datapath presented in this article based on validated and prioritized events. OpCode, Rs, Rt, ID_ReadData2_RF signals are signals from the pipeline to write to the nHSE scheduler registers mapped to the address space of the corresponding COP2 register file (RF). Reading and writing the preemptive scheduler registers is done using six instructions at COP2 level, namely CFC2 (copy control word from COP2), CTC2 (copy control word to COP2), LWC2 (load word to COP2 from data memory), SWC2 (store word to data memory in COP2), MFC2 (move control word from COP2) and MTC2 (move control word to COP2).

The nHSE_inhibit_CC signal prevents thread context switching when the CPU executes atomic write/read instructions to/from memory. Thus, the shared memory location should not be allowed to be accessed between reads and writes so that a race condition does not occur between processes. ExtIntEv[3:0] signals are used for asynchronous external interrupt events with the processor clock running at a frequency of 33 MHz. Finally, the clock signal is generated by the Xilinx^®^ LogiCORE™ IP Clocking Wizard 6.0 which is connected to the 200MHz differential clock signal (clock_200MHzP (E19 FPGA pin), clock_200MHzN (E18)) and the reset is connected to the RESET signal (AV40) of the Virtex-7 development kit. The synthesizable HW_nMPRA_RTOS implementation integrates a scheduler implemented in hardware to validate excellent performance at a more than the convenient cost in terms of FPGA resources used (Găitan & Zagan, 2021; Zagan & Găitan, 2022).

**Figure 3:**
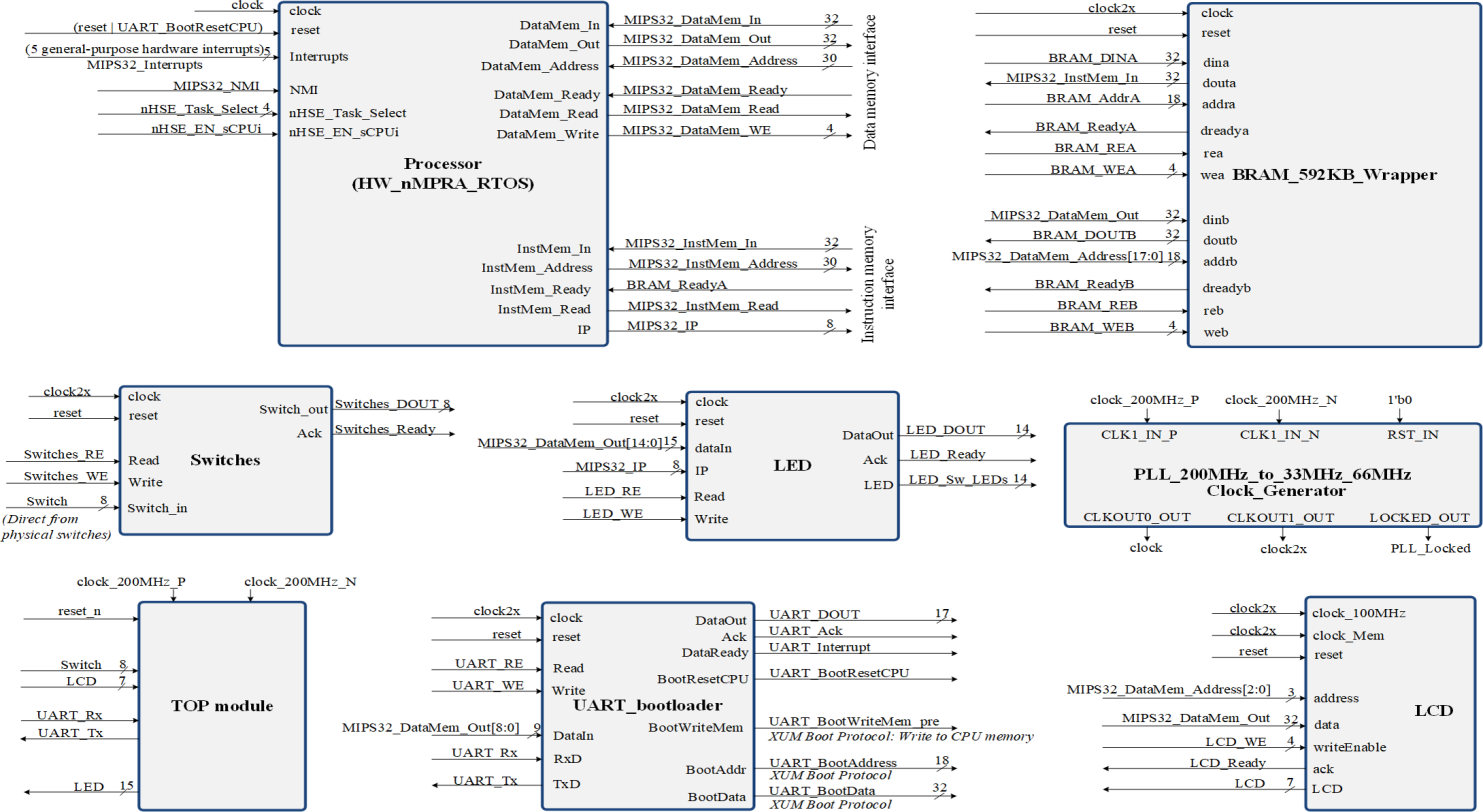
Structure of the SoC design containing the HW_nMPRA_RTOS processor.

Figure 3 represent a SoC design overview, showing inclusively the hazard detection modules, the CPU control unit and COP0. The Verilog TOP_Module connects to the clock signals and pins of the FPGA circuit (Ayers, 2020). Once all the modules were designed and tested, the Top module was created where all the blocks in the project with the corresponding logic are connected. Top.v represents the Verilog HDL file located at the highest level in the HW_nMPRA_RTOS project. It is also known as a motherboard that connects modules such as the CPU, memory, clock signals and I/O devices. All inputs and outputs, such as clock signals or UART transmit and receive pins, must match the pins of the FPGA circuit used. The Verilog Processor.v file together with the instantiation of the modules inside this file creates a complete MIPS32 processor. The high-level module is the Processor, and its interface consists of five general-purpose hardware interrupts, one non-maskable hardware interrupt, 8 diagnostic interrupts, and a dual-port memory interface implemented on-chip using IP Block Memory Generator 8.3 for both instructions and data. The processor module is the most important instantiated module in the HW_nMPRA_RTOS design. This file contains for the most part, the instantiation and linking of the basic processor blocks according to the design schematics. This module includes very little logic, although it contains most of the instantiated modules. In terms of boot procedure, the uart_bootloader module represents a standard hardware line connected with a bootloader for data transmission (Ayers, 2020). The LCD module represents the top-level interface to the display.

**Figure 4:**
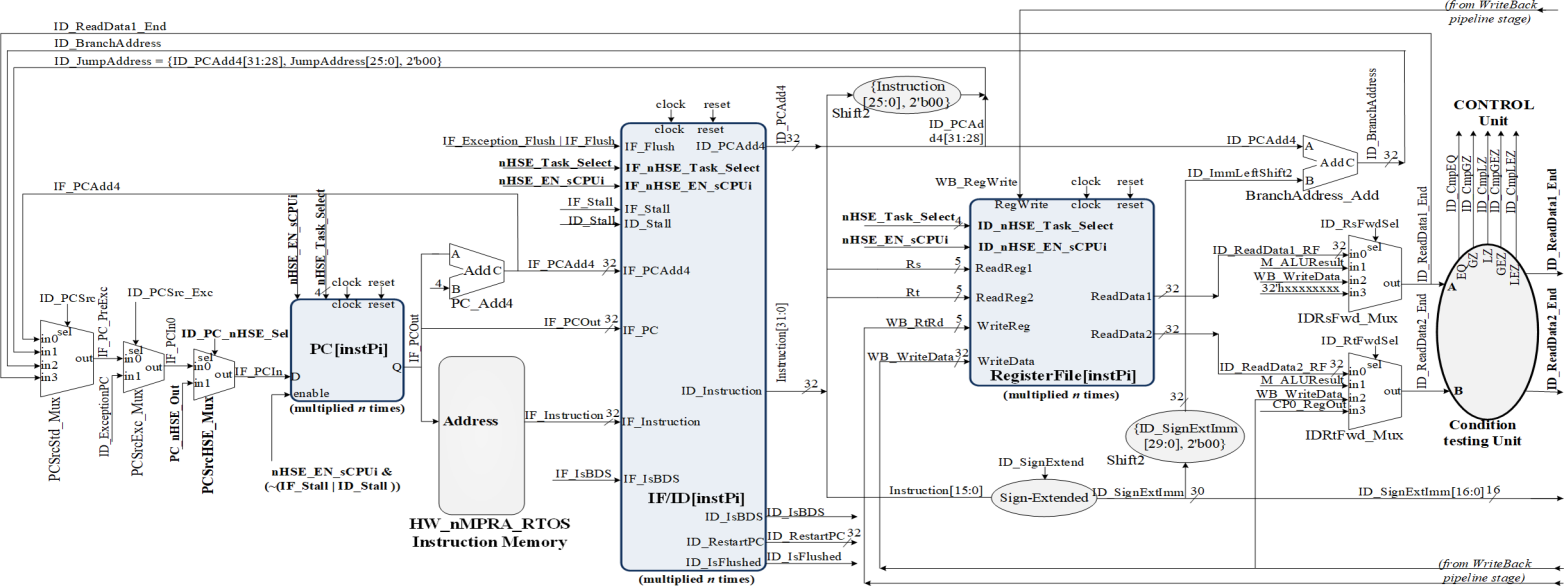
Pipeline stages implementation for instruction extracting and decoding based on HW_nMPRA_RTOS concept.

**Figure 5:**
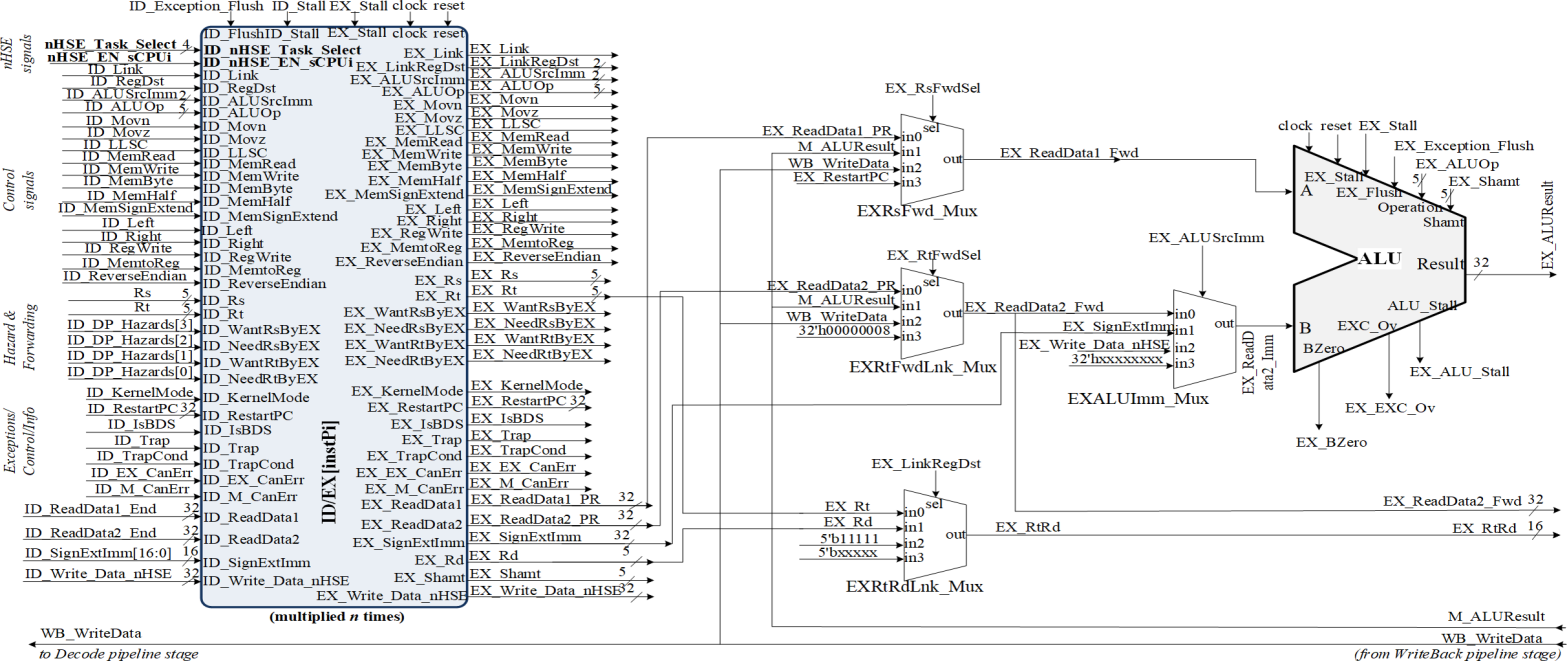
Implementation of the pipeline stage for execution and datapath resource multiplication in correspondence with HW_nMPRA_RTOS register-transfer level (RTL).

**Figure 6:**
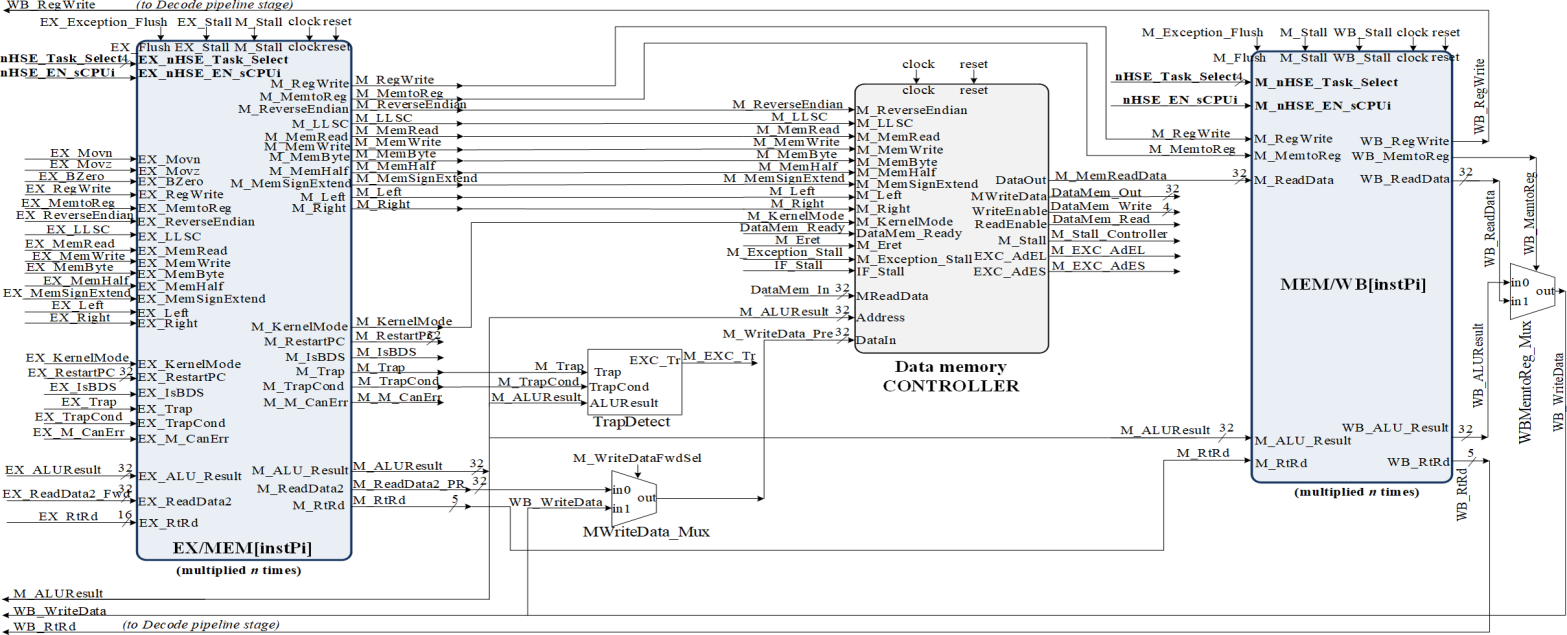
MEM and WB pipeline stages RTL implementation based on HW_thread_i multiplication resources.

As can be seen in Fig. 4, HW_nMPRA_RTOS relies on multiplying the resources in the datapath for each CPU instance, called HW_thread_i (Table 1). Thus, the notation PC[instPi] refers to the fact that the PC register is multiplied for each sCPUi (instPi), with i taking values from 0 to n-1, n being the maximum number of HW_thread_i chosen in the soft-core FPGA implementation. Multiplied resources have the same inputs, but the outputs are multiplexed internally according to i. For this reason, and to simplify the scheme the outputs have not been indexed (there is only one output depending on i). For the PC, RF, IF/ID, ID/EX, EX/MEM, and MEM/WB (Ayers, 2020) pipeline registers shown in Figs. 4, 5 and 6, their multiplication for each processor instance is indicated by the notation [instPi] (blurred blocks), the combinational structure being similar. Multiplication of pipeline registers was proposed and patented in Dodiu & Gaitan (2013).

The MIPS32 and MIPS64 instruction set architecture define a compatible 32-bit and 64-bit family within the global MIPS architectures. The MIPS32 architecture defines the following registers:

• A PC that is only indirectly affected by certain instructions and is not an architecturally visible register.
• General purpose working registers: 32-bit GPRs. Two of these registers have special functions, and register r0 is always zero and is hardware tied to logical zero (32′h00000000). This register can be used as a destination for any instruction whose result is to be discarded, or as a source when a null value is required. Register r31 is the default destination used by the JAL, BLTZAL, BLTZALL, BGEZAL, and BGEZALL instructions, but can also be used as a normal register.
• A pair of special registers named HI and LO, needed to store the result of multiplication and division operations. During the multiplication operation, the HI and LO registers store the product of the multiplication, and for division, the HI and LO registers to store the quotient (in HI) and the remainder (in LO) respectively.

In the IF (Instruction Fetch) stage together with the IF/ID (Instruction Fetch/Instruction Decode) pipeline register, the PC register is loaded with the address corresponding to the instruction in program memory to be fetched and then executed in the pipeline next stages. The PC register update is performed with one of the following addresses from the current stage or ID stage:

• IF_PCAdd4: output of the PC_Add4 adder;
• ID_JumpAddress: 32 bits representing {ID_PCAdd4[31:28], Instruction[25:0], 2′b00} for J-type instructions;
• ID_BranchAddress: conditional jump address {14{immediate[15]}, immediate, 2′b0};
• ID_ReadData1_End: address provided by the output of the IDRsFwd_Mux multiplexer in the ID stage.

The setting of the control signals for the multiplexers PCSrcStd_Mux (for PC source selection) and PCSrcExc_Mux (for PC exception selection) is performed by the HW_nMPRA_RTOS processor control unit and the CPZero module implementing coprocessor 0. In this stage there is also the PC_Add4 adder needed to add by four the current PC, relieving the arithmetic and logical unit of this operation. Thus, the IF/ID pipeline register will store the instruction fetched from program memory, the current PC value required for restart in case of an exception occurring in the next pipeline stages, and the PC+4 value required for fetching the next instruction. The IF_Stall, ID_Stall, IF_Exception_Flush, and IF_Flush signals are required by the control unit and the CPZero module (Platzer & Puschner, 2021), allowing stalling and flushing of the pipeline in case of hazard situations and exceptions. Operands read from the GPR will be stored in the next pipeline stage if the instruction is of type R or I, or will be ignored as is the case for jump instructions. Figure 4 illustrates the PCSrcStd_Mux and PCSrcExc_Mux multiplexers and the 32-bit outputs provided by these combinational circuits. In the ID pipeline stage, displacement registers are also designed for 32-bit word-level memory alignment, and the sign extension unit is designed to ensure data word width. As we can see in Fig. 4, this stage contains both the adder required for the calculation of jump addresses and the condition comparison unit. This unit has as inputs the two operands read from GPR and the output provides the logical conditions destined for the control unit. Figure 5 illustrates the pipeline execute (EX) stage and the pipeline ID/EX register.

One can see the connections between the redirection multiplexers for data hazard situations (EXRsFwd_Mux, EX_RtFwdLnk_Mux), the EXALUImm_Mux multiplexer for secondary operand selection, the EXRtRdLnk_Mux multiplexer for destination selection and the ALU unit. Figure 5 shows some of the signals contained in the ID/EX pipeline register, which is the largest resource consumer among the pipeline registers. Also illustrated are the operations provided to the arithmetic and logical unit, the ID_AluOp register, and the result of the required operation performed in the MEM and WB pipeline stages. The transmission and storage of the control signals through the datapath are performed concurrently with the data required for the execution of the operation dictated by the instruction opcode, thus guaranteeing the consistency of the contexts for an eventual change of the selected HW_thread_i.

Executing the code loaded *via* the Boot.coe file will test the datapath by observing the corresponding waveforms. Figure 5 depicts the signals generated by the HDU to signal the occurring hazard situation, the data forwarding unit selecting *via* the EX_RsFwdSel and EX_RtFwdSel signals the source of the operands in case of the encountered hazard. It can be seen that the EX_ALUResult register contains the result of the operation performed, while the EX_EXC_Ov register may indicate an overflow exception (Meakin, 2010). Note the variation of the EX_AluSrcImm selection signal for the EX-ALUImm_Mux multiplexer.

Figure 6 shows the implementation of the MEM and WB pipeline stages, thus completing the datapath for MIPS32 ISA-based processor with the HW_nMPRA_RTOS extension placed in COP2. It can be seen the propagation of the control signals and data redirection from the MEM pipeline stage. The data-saving operation can be noted when a register is copied from nHSE (COP2) to a general purpose register at the instPi level enabled *via* the nHSE_Task_select[3:0] and nHSE_EN_sCPUi signals.

**Figure 7:**
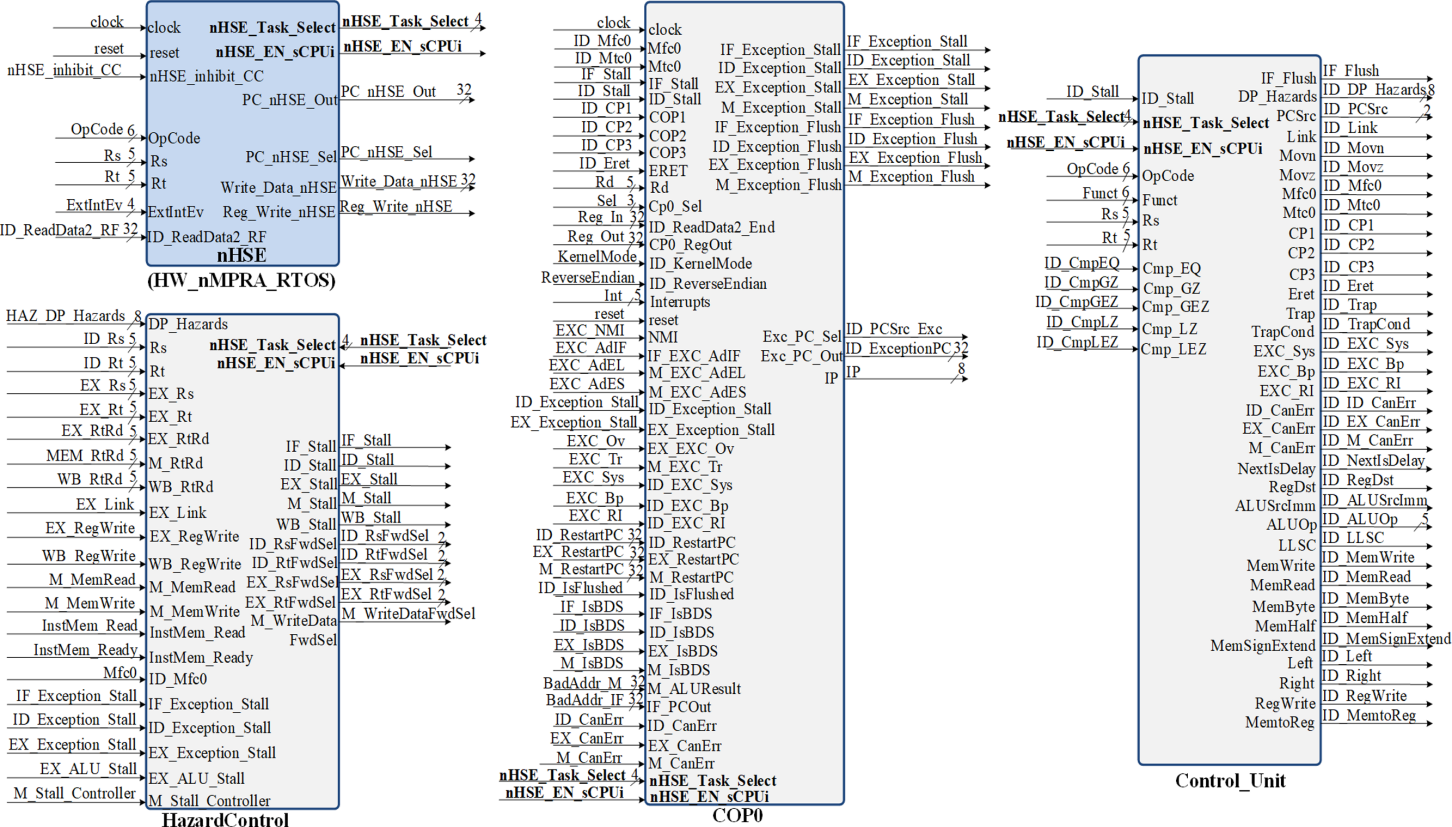
Block diagrams for hazard detection unit, nHSE, COP0 and control unit.

In the CPU implementation, control unit offer support for a flexible and high performance processor architecture. Figure 7 shows the inputs and outputs of the Control module (Ayers, 2020). The signals it outputs represent control lines and register type exceptions for the datapath as well as the operation passed to the arithmetic and logic unit. The control signals generation for the datapath occurs in the ID pipeline stage. The nHSE module illustrated in Fig. 7 is designed to satisfy the following architectural constraints:

• Preemptive scheduling of tasks and therefore interrupts type events;
• Implementation and management of time related events;
• PC selection;
• Select HW_thread_i *via* nHSE selector;
• CPU pipeline registers selection corresponding to each instPi.

The COP0 module shown in Fig. 7 represents MIPS32 Coprocessor 0. This module implements the processor management unit that allows the use of interrupts, trap cells, system calls as well as other exceptions. Distinction is made between user mode and kernel mode, providing status information with the ability to override program flow. This processor is designed for bare-metal memory accesses, therefore it cannot have virtual memory. However, the COP0 coprocessor subset complies with the MIPS32 architecture specification. Exceptions can occur in any pipeline stage, implying that more than one exception can be handled in a single cycle. When this happens, only redirection exceptions from the MEM stage to the EX stage are handled. The HazardControl module implements hazard detection and data redirection, allowing the HW_nMPRA_RTOS processor to operate correctly in the presence of data, structural and control hazards. This module detects if the current instruction requires data that is present in the HW_thread_i pipeline registers and needs to be forwarded or if the pipeline needs to be stalled. Most instructions read from one or more registers, and normally this happens in the instruction decode stage. However, accessing the GPR from the ID stage is slowed down when one or more stages in the HW_thread_i pipeline, such as EX, MEM, or WB, contain instPi instructions that perform an eventual write to the GPR but have not yet done so. The Control_Unit module is the control unit of the HW_nMPRA_RTOS processor. This unit sets the control signals in the datapath for each instruction read from memory. The signals depend on the executed instruction fields, the results of the condition test unit, and the ID_Stall signal provided by the hazard detection unit. Thus, the control signals accompany the instruction through each HW_thread_i pipeline stage, determining all necessary states and operations that the nMPRA processor must execute sequentially for each pipeline stage. Depending on CPU control signals and nHSE logic, Control_Unit sets the control bits required to execute each instPi instruction.

Branch detection options that are based on mutual exclusion (Branch_EQ, Branch_GTZ, Branch_LEZ, Branch_NEQ, Branch_GEZ, Branch_LTZ), cover portions of datapath that are not directly controlled by status signals. Note that these bits are part of the OpCode field of the instruction or other fields, representing an abstracted image of the instruction encoding. When new instructions are introduced, designers must ensure that they do not generate false information in the control bit status. In the MIPS architecture, jump and branch instructions have a delay slot, which means that the instruction following a jump or branch is executed before the jump or branch occurs. MIPS processors execute the jump or branch instruction and the delay slot instruction as an indivisible unit. If there is an exception as a result of the execution of the delay slot instruction, the jump or branch instruction is not executed and the exception appears to be caused by the jump or branch instruction.

Within the HW_nMPRA_RTOS processor, all jump and branch operations cause the execution of the instruction in the branch delay slot, regardless of whether the branch is performed or not. Exceptions related to jump instructions are part of the branch likely instruction group and are not implemented in the control module. In addition to this, there is a group of conditional jump instructions, called branch likely, for which the next instruction that is in the so-called delay slot is executed only if branching occurs. Even though branch likely instructions are included in the MIPS specification, the software is encouraged to avoid these instructions as they will be removed from future revisions of the MIPS architecture. Therefore, the branch likely conditional jump instructions (BEQL, BGEZALL, BGEZL, BGTZL, BLEZL, BLTZALL, BLTZL, BNEL) have not been implemented in the HW_nMPRA_RTOS soft-core processor. For the datapath corresponding to the HW_nMPRA_RTOS processor, all signals are active on 1L. The jump and branch lines determined by PCSrc as well as those determined by the arithmetic and logical unit operation are handled by the control unit.

**Figure 8:**
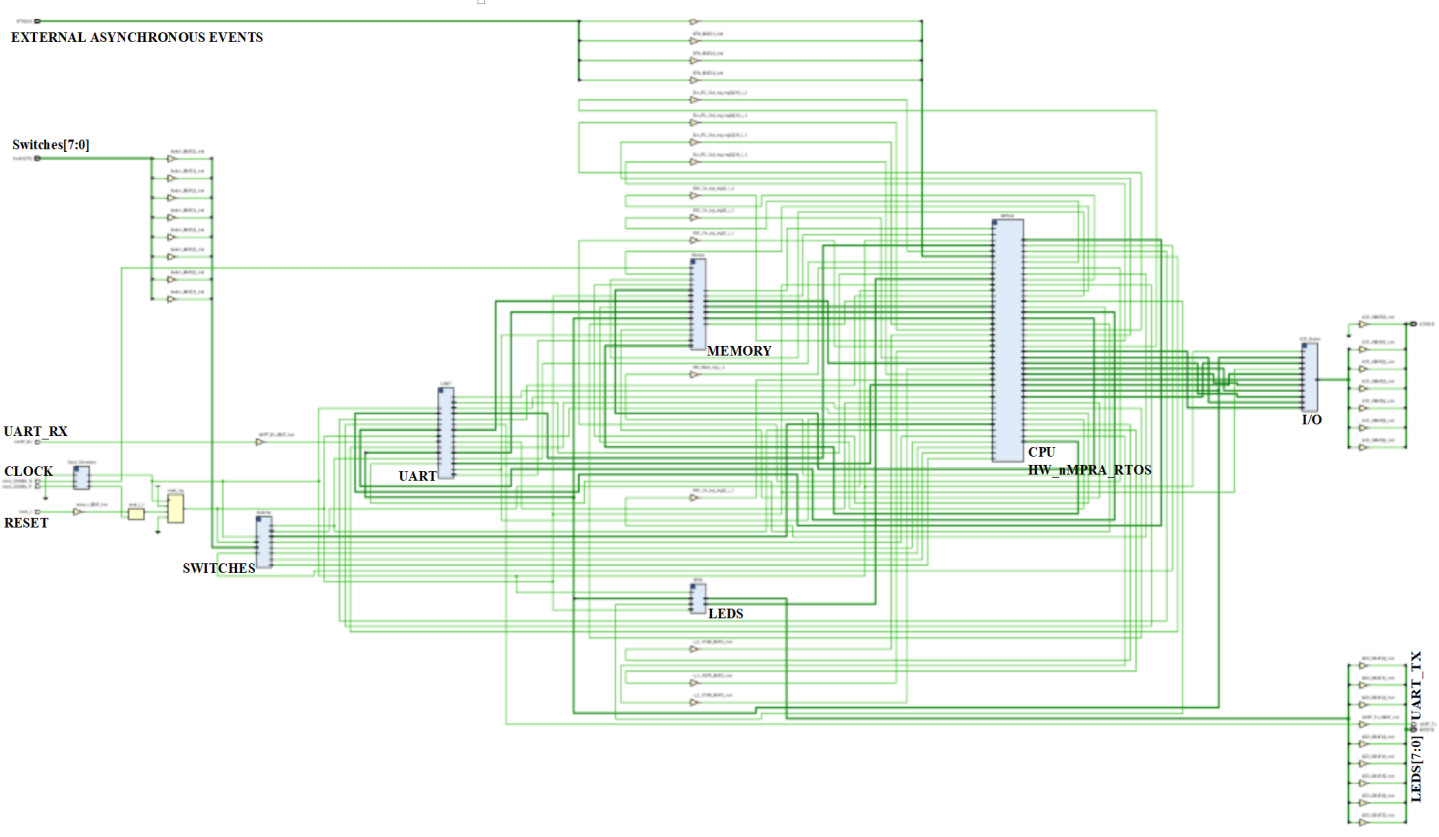
Custom soft-core processor FPGA implementation after synthesis in FPGA using Vivado DS custom soft-core processor FPGA implementation after synthesis in FPGA using Vivado DS.

In Găitan & Zagan (2021), instructions dedicated to the control of the HW_nMPRA_RTOS integrated scheduling unit are described. Its behavior is controlled *via* a dedicated instruction set, supporting dynamic interrupt management mechanisms and power-safe functions. The HW_nMPRA_RTOS processor RF contains HW_thread_i \time 32 general purpose registers of 32 bits each and two read ports for them depending on the selected task. Figure 4 shows the RF for HW_nMPRA_RTOS processor based on HW_thread_i multiplication. Register 0 is always set to the value 32′h00000000. Writing to the RF is performed according to the semi-processor selected by the hardware scheduler. At the positive clock edge, the data provided by the WriteData input (32 bits) is written to the WriteReg index register (5 bits) on command of the RegWrite signal. The combinational read from the RF is based on the scheduled task ID_nHSE_Task_Select. Figure 8 shows the RTL schematic generated by the Xilinx Vivado DS software after synthesizing the HW_nMPRA_RTOS processor. This contains all the blocks instantiated in the SoC Top.v module using Verilog HDL.

### ARM Cortex-M4 experimental findings

This subsection presents practical tests performed using Cortex-M4 and a software RTOS to make a comparison with the hardware RTOS implemented on HW_nMPRA_RTOS. The overall objective of this article is to present the main issues related to RISC CPU types, considering their use in embedded system design and implementation. The biggest advantage of microcontrollers over microprocessors relates to design and hardware costs which are much lower and can be kept to a minimum. Cortex-M4 processors feature a configurable interrupt controller that can support up to 240 vectored interrupts and multiple levels of interrupt priority (from 8 to 256 levels). Interrupt nesting is handled automatically by hardware, interrupt latency is only 12 clock cycles for memory systems with zero wait states.

The interrupt-handling capability makes Cortex-M processors suitable for many real-time applications (Yiu, 2019). The Cortex-M4 processor contains all the features of the Cortex-M3 processor, has additional instructions to support DSP applications, and has the option to include a floating point computing unit (FPU).

Cortex-Mx processors have a simple, linear memory map, with the same system-level and debugging features as the Cortex-M3 processor. There are no special architectural restrictions that can often be found in 8-bit microcontrollers (*e.g.*, bank-organized memory, limited stack levels, non-reentrant code, etc.). Designers can program almost everything in C, including the interrupt handler. ARM Cortex-M4 processor allows for 240 interrupt requests (IRQ), priorities being programmable by the user, with the exception of non-maskable interrupt (NMI) and HardFault which have fixed priorities. Nested Vector Interrupt Controller (NVIC) is used to dynamically decide which interrupt is more important and to enable or disable them. NVIC supports up to 256 different interrupt vectors. In the following, we present the jitter measurement corresponding to the occurrence of an external asynchronous signal related to an embedded device based on a RISC architecture.

**Figure 9:**
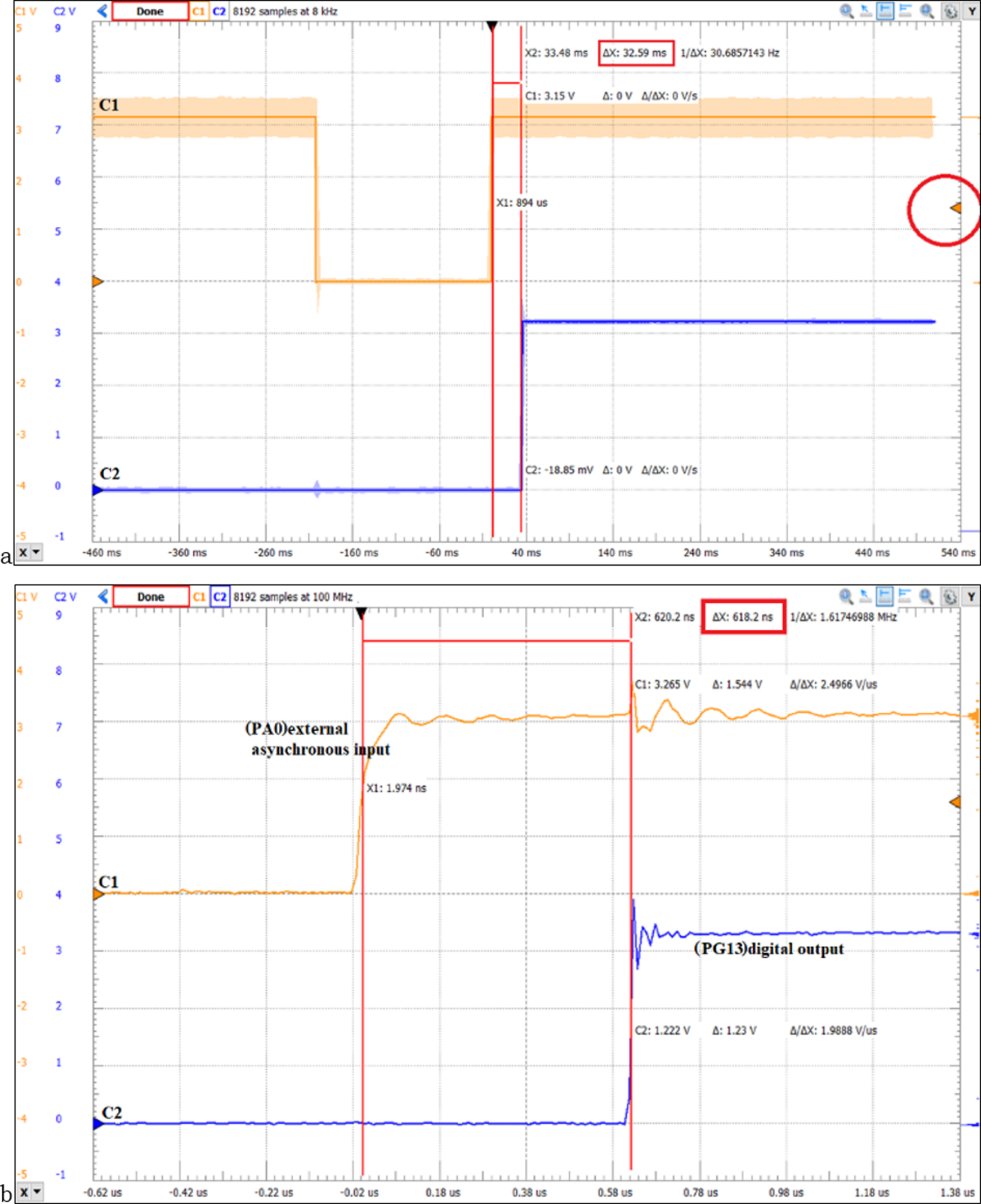
(A) STM32F429ZIT Cortex-M4 MCU, WaveForms software and ANALOG2 DISCOVERY DIGILENT oscilloscope to measure response time for program data transfer (32.59 ms); (B) SoC design timing report based on Xilinx FPGA and Vivado DS.

Using the STM32F429ZIT microcontroller based on Cortex-M4 architecture, FreeRTOS, DIGILENT DISCOVERY ANALOG2 oscilloscope, and WaveForms software, the jitter is measured in case of external interrupt handling. The asynchronous event with the ARM processor is in accordance with the falling edge of the signal connected to the PA0 pin, measuring the period of time until the state of the PG13 pin which controls an LED changes (the EXTI0_IRQHandler executes the instructions corresponding to the LD3 led (BSP_LED_Toggle(LED3)). Figure 9 illustrates the WaveForms software for measuring the real-time response time using NVIC (200 ns/div will be set to time). The Channel C1 signal represents the PA0 digital input connected to the USER button, the oscilloscope trigger is set on the rising edge (EXTI_InitStruct.EXTI_Trigger = EXTI_Trigger_Rising). Oscilloscope Channel C2 displays the signal corresponding to pin PG13 (LD3), its state is changed by executing GPIO_WriteBit(LED1_Port, LED1_Pin, Bit_SET) function. In the case of the measurements in Fig. 9A software filtering of the PA0 input signal was also performed. It should be noted that Cortex-M4 RISC processors have a three-stage pipeline design and a Harvard bus architecture with unified memory space for instructions and data. For the case of using external interrupt and NVIC we obtained a response time of 618.2 ns (Fig. 9B) and for the case of using program data transfer, we obtained a response time of 32.59 ms (Fig. 9A).

### HW_nMPRA_RTOS hardware scheduler implementation setup

To improve real-time performance and minimize RTS jitter, the preemptive scheduler in the new HW_nMPRA_RTOS component implements in hardware the logic for handling events attached to each instPi. In the nHSE scheduler, external interrupt events inherit the priority of the instPi CPU instance to which they are attached, thus guaranteeing the required deadlines. Table 2 present the application sequence program used for experimental testing. Thus, the corresponding HW_thread_i datapath was validated with preemptive instPi scheduling. The instP3 and instP0 instances were executed correctly by checking the instructions and the corresponding addressing modes. The stmr instruction is of type MFC2 (move monitoring/control word from COP2) and has opcode 0100_10 and rs field 00000. Instructions of this type in Table 2 (48060000 h, 48020000 h, 48430001h) are intended to copy nHSE scheduler registers, such as mrCntRuni, mrTEVi and crEVi, to the GPR. The movcr instruction (48C10000h) is of type CTC2 with opcode 0100_10, rs field 00110, and has the effect in the case of the presented program to update the crTRi register. The sw instruction (ADCC0000h) updates the outputs corresponding to the LEDs, which are mapped in the address space of the data memory (MemAddr[29:26] = 4′b1100), as can be seen in the experimental tests, namely in the measurement of response time to an asynchronous CPU event.

**Table 2:** The application sequence program used for experimental testing.

MIPS32 instructions (included COP2)	ID_Instruction [31:0] signals (machine code)	Application description
…		instP3 execution on HW_thread_3 scheduled by nHSE
stmr	48060000 h	Instruction stmr (store monitoring register): copy from register mrCntRuni[instP3] COP2 in r6 (GPR)
stmr	48020000 h	Instruction stmr: copy from register mrTEVi[instP3] COP2 in r2 (GPR)
addi	20010071 h	Add Immediate: R[rt=1]=R[rs=0]+SignExtImm
addiu	24420001 h	Add Immediate Unsigned: R[rt=2]=R[rs=2]+SignExtImm
stmr	48430001 h	Instruction stmr: copy from the register crEVi[instP3] in r3 (GPR)
addi	20010011 h	Add Immediate: R[rt=1]=R[rs=0]+SignExtImm (=0011)
movcr	48C10000 h	Instruction movcr (move control register) (*wait Rj* instruction): copy from register r1 (GPR) in crTRi[instP3] COP2 and determines the context switch if instP0 has no validated and active events
…		No instPi in execution
…		instP0 execution on HW_thread_1 (scheduled for handling an external interrupt type event (ExtIntEv[0]) according to the algorithm in Table 3)
addi	20010000 h	Add Immediate: R[rt=1]=R[rs=0]+SignExtImm (=0000)
addi	200E0003 h	Add Immediate: SignExtImm = 0003, rd = r14
sll	000E7780 h	Shift left logical: Shamt = 30, rd = r14 (I/O LED: MIPS32_Data_IO_MemAddr[29:26] = 4′b1100)
addi	200C00F0 h	Add Immediate: SignExtImm = 00f0, rd = r12, write the value 32′h000000f0 in r12 (GPR)
sw	ADCC0000 h	Store word: save r12 (GPR) to the address stored in r14
movcr	48C10000 h	Instruction movcr (*wait Rj* instruction): causes the instP0 dispatch if there are no other active (crEVi) and validated (crTRi) events for instP1

Following the tests, the nHSE hardware scheduler specification was revised and the synthesis and mapping stage in FPGA using Vivado was performed.

Table 3 shows the logic for selecting the interrupt event and assigning it to the instPi for preemptive execution. The HW_nMPRA_RTOS registers are described extensively in the processor specification and in Găitan & Zagan (2021).

**Table 3:** Algoithm: Interrupt event handling implemented in nHSE scheduler hardware (HW_nMPRA_RTOS processor).

1:	**always** @(posedge clock) **begin**
2:	case (nHSE_FSM_state)
3:	FSM_WAIT: **begin**
4:	nHSE_EN_sCPUi <= DISABLE;
5:	**end**
6:	FSM_sCPU0: **begin**
7:	nHSE_sCPUi_Select <= sCPU0_ID;
8:	nHSE_EN_sCPUi <= (cr0MSTOP & Mask1_bit0) ? ENABLE: DISABLE; // sCPU0 ready?
9:	if(((crTRi[sCPU0_ID] & Mask1_bit4)&&(crEVi[sCPU0_ID] & Mask1_bit4))&&(∼((crEPRi0[17:15] <crEPRi0 [14:12])&&((crTRi[sCPU0_ID] & Mask1_bit5)&&(crEVi[sCPU0_ID] & Mask1_bit5)))— ((crEPRi0[20:18] <crEPRi0[14:12])&&((crTRi[sCPU0_ID] & Mask1_bit6)&&(crEVi[sCPU0_ID] & Mask1_bit6))))) **begin** // external interrupt handling
10:	for(i_INT0=NR_INT-1;i_INT0>=0;i_INT0=i_INT0-1) **begin**
11:	if((grINT_IDi[i_INT0] == sCPU0_ID)) **begin**
12:	if(grEv_select_sCPU[sCPU0_ID] == 3′b111) **begin**
13:	grEv_select_sCPU[sCPU0_ID] <= 3′b100; //the position of the event generated from crEPRi
14:	crEVi[sCPU0_ID] <= crEVi[sCPU0_ID] — 32′h00000080;
15:	crTRi[sCPU0_ID] <= crTRi[sCPU0_ID] — 32′h00000080;
16:	if(grInt_select_sCPU[sCPU0_ID] == 8′hFF) begin
17:	PC_nHSE_Out <= EXC_Vector_Base_INTi[i_INT0]; // jump to trap cell for interrupt *i*
18:	PC_nHSE_Sel <= 1′b1; //select the following value for the PC register
19:	grInt_select_sCPU[sCPU0_ID] <= i_INT0; //save the occurrence of the interruption event
20:	Inhibit_Context_Switch <= 1′b0;
21:	**end**
22:	**end**
23:	else **begin**
24:	PC_nHSE_Sel <= 1′b0;
25:	if((Reg_Write_nHSE_WB==1)&(WB_OpCode==Op_Type_CP2) & (WB_Rs==Rs_Type_CTC2) & ((WB_Immediate == 16′h0001) & ((Write_Data_WB & 32′h00000080)== 32′h00000000))) **begin**
26:	grInt_select_sCPU[sCPU0_ID] <= 8′hFF;
27:	grEv_select_sCPU[sCPU0_ID] <= 3′b111;
28:	**end**
29:	**end**
30:	**end**
31:	**end** //for
32:	**end** //if interrupts
33:	**end**
34:	**endcase**
35:	**end** //always

At each rising edge of the clock signal, the finite state machine (FSM) checks the current state of the scheduler by testing nHSE_FSM_state variable. When the FSM is in the FSM_WAIT state then the scheduler does not execute any instPi and the nHSE_EN_sCPUi signal is disabled (Fig. 2). If FSM is in FSM_sCPU0 state then instP0 is currently executing (nHSE_sCPUi_Select <= sCPU0_ID) and will test the associated events. The condition for instP0 to handle an external interrupt event is given by Table 3, line 9. Therefore, the prioritization of all events is done at the level of each instPi through the crEPRi register (Table 1). The crTRi[sCPUi_ID] control register validates time, deadline, interrupt (Table 3, line 19), mutex, and synchronization events, and the crEVi[sCPUi_ID] register indicates their occurrence. The algorithm tests if an interrupt event has occurred that is attached to instP0 (grINT_IDi[i_INT0] = = sCPU0_ID) and jump to the trap cell for interrupt i_INT0. Next, set the PC_nHSE_Sel line (Fig. 4) to select the following value for the PC register. Otherwise, it is indicated that no event is currently handled by nHSE (grEv_select_sCPU[sCPU0_ID] <= 3′b111).

The condition in line 31 of the algorithm tests whether the current instruction is an instruction of type Op_Type_CP2 and whether it is written in COP2 (WB_Rs = =Rs_Type_CTC2). Thus, the code in Table 3 handles an interrupt event assigned to a processor instance (instP0), prioritized *via* the crEPRi register, directly jumps to its associated trap cell, and ensures correct FSM operation without generating a race condition. Table 2 presents a benchmark program used for experimental testing, so that through simulations and practical measurements with the oscilloscope can measure the time to capture the external event, the time to change the state of the finite state machine and the context switch.

## Results & Discussion

The main technical objective of this concept is to design and integrate HW_nMPRA_RTOS for a SoC with predictable time behavior and real-time response since the nHSE dynamic scheduler has a negligible implication on RTOS jitter. So, performing the previous tests with ARM Cortex-M4 and synthesizing in FPGA the Microblaze soft-core, this subsection presents the HW_nMPRA_RTOS concept FPGA integration and the interrupt events response time measurement.

**Figure 10:**
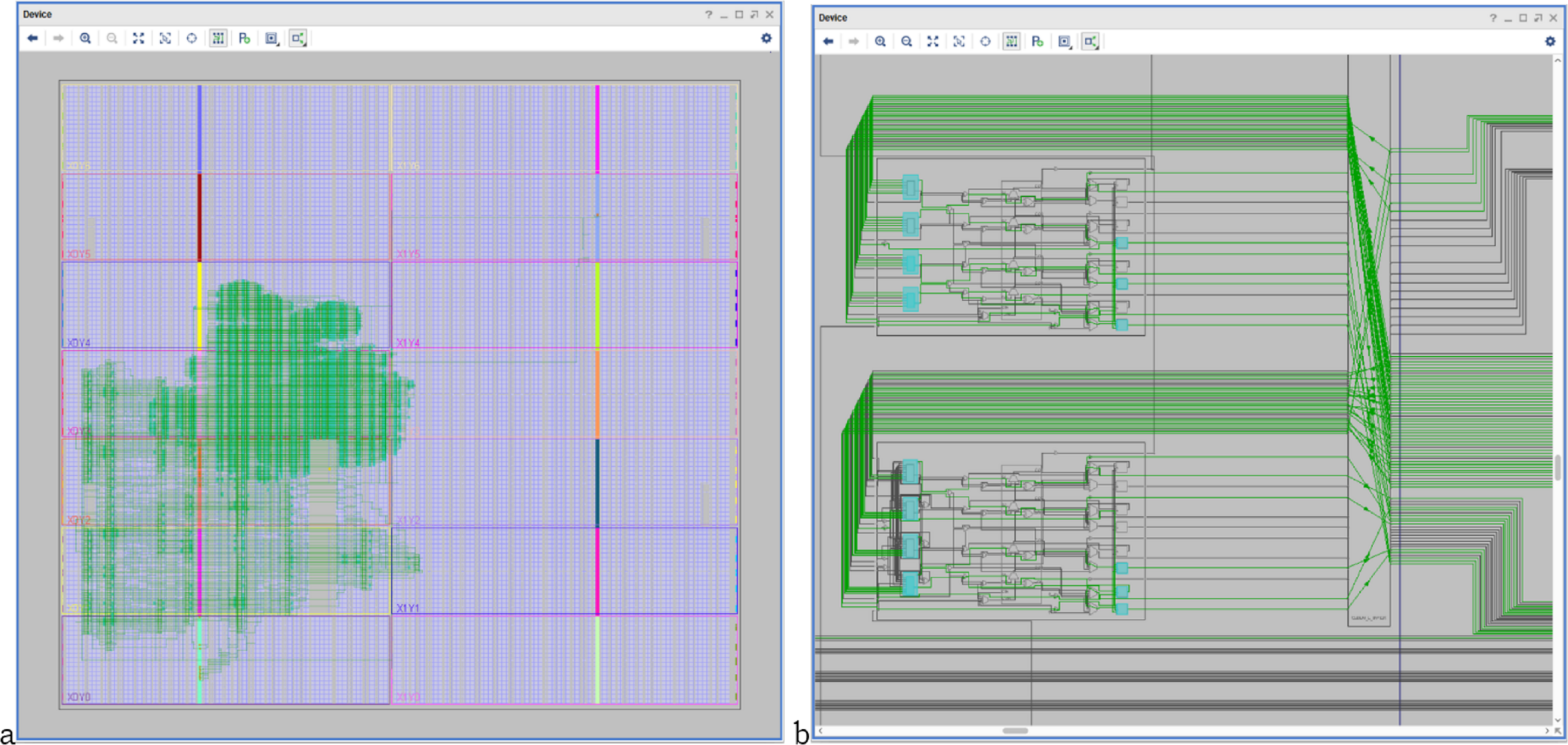
(A) HW_nMPRA_RTOS design optimized for 28 nm system performance and FPGA integration; (B) HW_nMPRA_RTOS soft-core placement and layout in Xilinx Virtex-7 FPGA VC707 evaluation kit based on XC7VX485T-2FFG1761C circuit.

Figure 10 shows the HW_nMPRA_RTOS design synthesis and SoC integration on 28 nm technology (Gary et al., 2017), as well as the soft-core CPU placement and layout in the Xilinx Virtex-7 FPGA VC707 evaluation kit based on XC7VXX485T-2FFG1761C circuit. Based on the multiplication of resources in the datapath and the preemptive scheduler implemented in hardware, thread contexts switching in HW_nMPRA_RTOS is performed in a maximum of 1 ÷ 2 clock cycles (maximum of 60.6 ns at a frequency of 33 MHz). Thus, in the case of a software RTOS, the switching of thread contexts takes place in a few microseconds. From a safety-critical application point of view, the HW_nMPRA_RTOS architecture represents an innovative, low-cost solution (including RTOS) with better performance than existing systems in automotive, robotics, medical, motion control, monitoring, and control of fast and slow processes.

 This achieves minimal jitter in handling interrupts and real-time tasks, ensuring the stability of critical RTSs controlled by HW_nMPRA_RTOS.

Table 4 shows the Artix-7 FPGA resource requirements for MicroBlaze, Cortex-M3, RISC-V, MIPS32 and HW_nMPRA_RTOS FPGA implementation architectures (Włostowski, Serrano & Vaga, 2015; Li, Zhang & Bao, 2022; Tsai & Lee, 2022; Sarjoughian, Chen & Burger, 2008). It can be stated that the flip-flops and combinational logic requirements are convenient for the architecture proposed in this article, considering that HW_nMPRA_RTOS guarantees context switching in 1 ÷ 2 clock cycles and predictable response to interrupt events.

**Table 4:** Post-implementation FPGA resource requirements for different CPU pipeline implementations (FPGA chip XC7A100T-1CSG324C).

XC7A100T (available resources)/ CPU	MicroBlaze	Cortex-M3	uRV RISC-V core (Włostowski, Serrano & Vaga, 2015)	HW_nMPRA_RTOS (4 HW_thread_i)	Ultraembedded RISC-V (Li, Zhang & Bao, 2022)	Aquila RISC-V SOC (Kintex-7 KC705) (Tsai & Lee, 2022)	MIPS32 (multicycle) (Sarjoughian, Chen & Burger, 2008)	MIPS32 (pipeline with cache) (Sarjoughian, Chen & Burger, 2008)
LUT (63400)	1512	22559	4242	15958	13170	30705	1751	3453
LUTRAM (19000)	224	6144	608	814	866	5666	136	16
FF (126800)	1389	6384	4047	8613	10002	31739	249	1908
BRAM (135)	8	–	513	148	5	36	–	–
DSP (240)	3	3	4	4	4	–	–	–
IO (210)	25	41	28	32	57	119	46	48
BUFG (32)	3	8	3	15	3	5	2	6
MMCM (6)	1	1	1	1	1	2	–	–

Table 5 shows the power consumption results for three FPGA implementations considered, namely MicroBlaze, ARM Cortex-M3, RISC-V, MIPS32 and HW_nMPRA_RTOS (with 4 HW_thread_i). Total on-chip power is represented by static and dynamic on-chip power which is also referred to as thermal power and includes on-chip dissipated power from any source. Static on-chip power is composed of the values sum obtained for static device and static design. The static device coefficient is represented by transistors leakage power when the device is powered and not configured, and design static (standby power) indicates the power when the device is configured and there is no switching activity, although it also includes static power in I/O digitally controlled impedance (DCI) terminations. Effective thermal resistance depends mainly on the heatsink and board characteristics, airflow and user selected package.

**Table 5:** Post-Implementation on-chip power results.

Artix-7 FPGA/ CPU	MicroBlaze	Cortex-M3	uRV RISC-V core (Włostowski, Serrano & Vaga, 2015)	HW_nMPRA_RTOS (4 instPi)	Ultraembedded RISC-V (Li, Zhang & Bao, 2022)	Aquila RISC-V SOC (Kintex-7 KC705) (Tsai & Lee, 2022)	MIPS32 (multicycle) (Sarjoughian, Chen & Burger, 2008)	MIPS32 (pipeline with cache) (Sarjoughian, Chen & Burger, 2008)
Total on-chip power	0.233 W	0.255 W	0.649 W	0.421 W	0.781 W	2.839 W	0.133 W	0.171 W
Static on-chip power	0.098 W (42%)	0.072 W (28%)	0.275 W (42%)	0.252 W (60%)	0.064 W	0.180 W	0.097 W	0.097 W
Dynamic on-chip power	0.135 W (58%)	0.183 W (72%)	0.374 W (58%)	0.169 W (40%)	0.717 W	2.659 W	0.036 W	0.073 W
Confidence level	Low	Low	Low	Low	Low	Low	Low	Low
Power supplied to off-chip devices	0 W	0 W	0 W	0 W	0.506 W	0.44 W	0 W	0 W
Effective thermal resistance	4.6 °C/W	4.9 °C/W	1.1 °C/W	1.1 °C/W	4.8 °C/W	1.8 °C/W	4.6 °C/W	4.6 °C/W
Thermal margin	58.9 °C (12.8 W)	73.7 °C (14.8 W)	59.3 °C (50.1 W)	59.5 °C (50.4 W)	71.3 °C	55.0 °C	59.4 °C	59.2 °C
Junction temperature	26.1 °C	26.3 °C	25.7 °C	25.5 °C	28.7 °C	30.0 °C	25.6 °C	25.8 °C

Thermal margin represent the temperature and power margin to or in excess of the maximum accepted range for the selected device grade. This value can be used to decide how best to address the excess power consumed on-chip. Junction temperature presented in Table 5 is an estimated value (Vivado DS), which is calculated up to the absolute maximum temperature after witch point 125+ is clearly highlighted and the power thermal estimates are no longer valid. Thus the total on-chip power is consistent with the hardware contexts implemented separately for instPi and also with the junction temperature, so we can say that the aim of this project has been successfully achieved.

**Figure 11:**
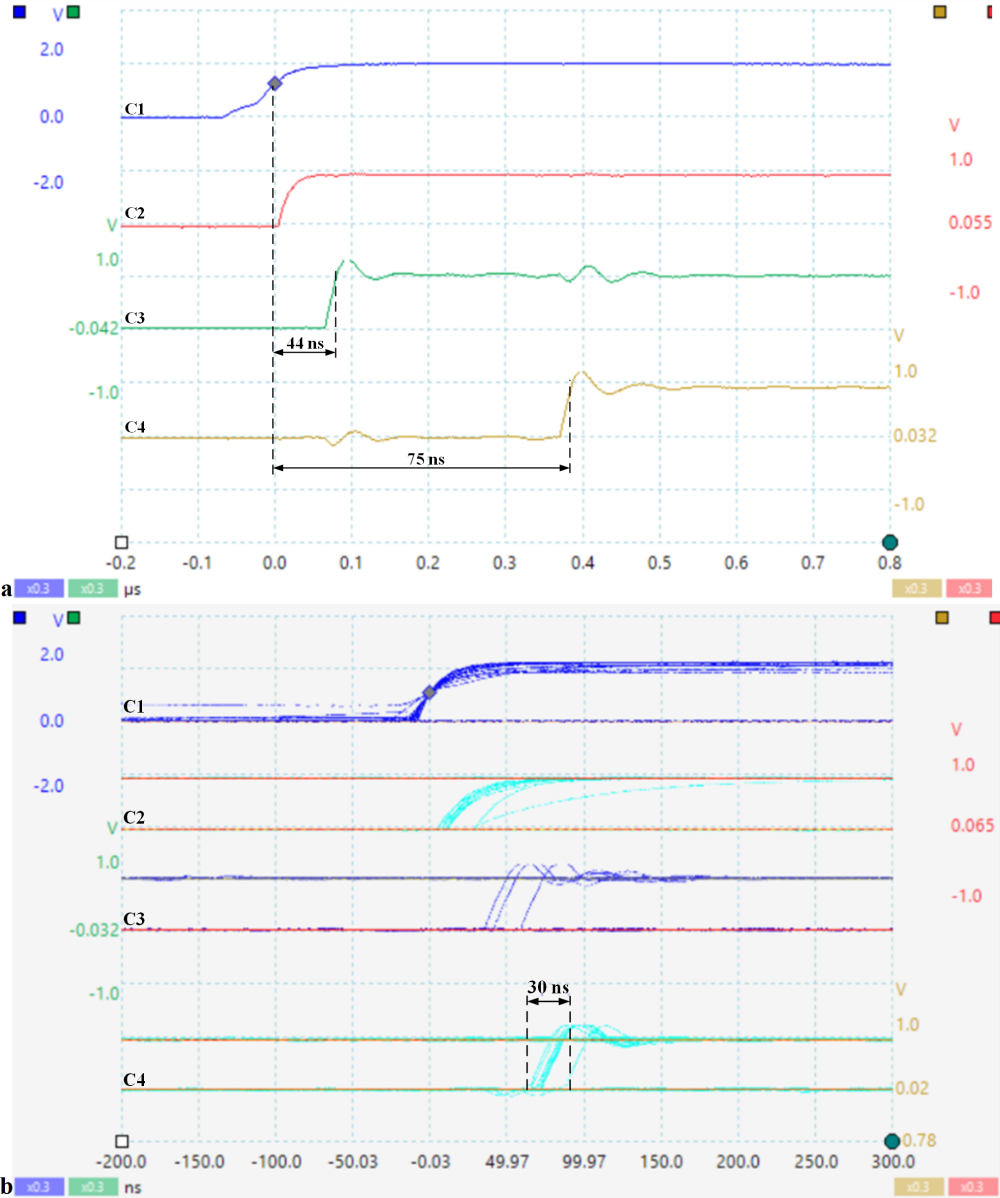
(A) Response time to an asynchronous CPU event; (B) Jitter corresponding to the tasks hardware scheduling (soft-core processor frequency is 33 MHz).

Figure 11 shows the response time to an asynchronous event and the jitter corresponding to scheduling a set of tasks in hardware. The signal captures are taken with PICOSCOPE 6404D oscilloscope (Pico Technology, St. Neots, UK) with four channels and max 500 MHz. Thus, channel C1 indicates the occurrence of the external asynchronous signal with the processor clock, C2 indicates the storage of this event by setting the evIi bit in the crEVi control register (Table 3), C3 indicates the FSM state (nHSE_FSM_state from Table 3), and C4 shows the selection of the appropriate task for execution by the scheduler (en_pipe_sCPU0).

As can be seen in Fig. 11A, the response time to a prioritized outage event is 75 ns, the measurement was performed from when the event occurred until nHSE switched instP0 to HW_thread_0. The jitter is a maximum of 30 ns (Fig. 11B), depending on when the input signal is captured and stored in the corresponding bit of register crEV0, this event being validated by the corresponding bit of crTR0.

In contrast to classical processor architectures, where context saving involves saving registers on the stack causing a jitter effect, the processor architecture described in this article ensures much-needed predictable behavior in critical situations. The size of the memory consumed for the implementation of the multiplied resources, PC register, GPR, and pipeline registers, is directly proportional to the number of implemented HW_thread_i. To meet the real-time requirements in the RTS domain, nHSE implements a priority-based scheduling scheme. Future research will consider implementing the earliest deadline first (EDF) scheduling algorithm in hardware.

## Conclusions

The synthesis and FPGA implementation of this project will facilitate the development of RTS applications. The research and proposals carried out for this work have been validated in practice and the scientific results have been compared based on well-chosen experiments. The scientific contribution and economic benefits of the proposed concept will imply significant increases in industrial products due to its easy integration into software applications of new RTS-based applications, and can even be integrated into a set of Building Internet of Things (BIoT)-based smart switches. To better evaluate the performance of the processor model addressed in this article, the most representative implementations in the field were considered in the implementation and performance analysis of RTOS. Following the presentation and description of the HW_nMPRA_RTOS architecture and the analysis of processor architectures with hardware-implemented functions we can deduce the following achievements:

• Studies on the HW_nMPRA_RTOS processor implementation involving the custom pipeline stages and GPR implementation (Figs. 3 ÷ 7);
• Comparative analysis of the most representative soft-core processor implementations (MicroBlaze), both hardware and software proposed in the current literature (Section 4.2);
• Integration, presentation and description of the nHSE scheduling results implemented, and validated in practice (Section 4.3 and 4.4).

The existence of dedicated HW_thread_i resources, *i.e.,* the ability to flexibly set instPi priorities and dynamically attach interrupt events, guarantees in addition to fast event response and robust priority-based preemptive scheduling.

##  Supplemental Information

10.7717/peerj-cs.1300/supp-1 Supplemental Information 1 Hardware Scheduler Engine for 4 instPi (VERILOG HDL file) Provides a language abstraction for the nHSE_MIPS32-specific op-codes and the processor-specific datapath, hazard, and exception bits which control the processor. These parameter names are used extensively throughout the processor HDL modules.

10.7717/peerj-cs.1300/supp-2 Supplemental Information 2 NHSE and MIPS32 CPU datapath control signals (VERILOG HDL file) Provides a language abstraction for the nHSE_MIPS32-specific op-codes and the processor-specific datapath, hazard, and exception bits which control the processor. These parameter names are used extensively throughout the processor HDL modules.

## Additional Information and Declarations

### Data Availability

The following information was supplied regarding data availability: The code is available in the Supplemental Files.

## References

[ref-1] AMD (2017). MicroBlaze Soft Processor Core. https://www.xilinx.com/products/design-tools/microblaze.html.

[ref-2] (2011). MIPS^®^ architecture for programmers volume I-A: introduction to the MIPS32^®^ Architecture, Revision 3.02, Mar. 2011. https://courses.engr.illinois.edu/cs426/Resources/MIPS32INT-AFP-03.02.pdf.

[ref-3] Aurora Dugo AT, Lefoul J-B, Ben-Salem A, Harnois S, De Magalhaes FG, Nicolescu G (2022). Efficient scheduling mapping and memory bandwidth allocation for safety-critical systems.

[ref-4] Ayers G (2020). eXtensible Utah Multicore (XUM) project at the University of Utah. 2011–2012. http://opencores.org/project,mips32r1.

[ref-5] Bae W (2021). Today’s computing challenges: opportunities for computer hardware design. PeerJ Computer Science.

[ref-6] Chen S, Liu J, Tu W, Li Z, Hu W (2019). A method of improving the performance of hybrid reconfigurable hardware.

[ref-7] Ciobanu E-E (2018). The events priority in the nMPRA and consumption of resources analysis on the FPGA. Advances in Electrical and Computer Engineering.

[ref-8] Coluccio A, Ieva A, Riente F, Roch MR, Ottavi M, Vacca M (2022). RISC-Vlim, a RISC-V framework for logic-in-memory architectures. Electronics.

[ref-9] Dantas LP, De Azevedo RJ, Gimenez SP (2019a). A novel processor architecture with a hardware microkernel to improve the performance of task-based systems. IEEE Embedded Systems Letters.

[ref-10] Dantas LP, De Azevedo RJ, Gimenez SP (2019b). A novel processor architecture with a hardware microkernel to improve the performance of task-based systems. IEEE Embedded Systems Letters.

[ref-11] Dodiu E, Gaitan VG (2013). Central processing unit with combined into a bank pipeline registers.

[ref-12] Doerflinger A, Albers M, Kleinbeck B, Guan Y, Michalik H, Klink R, Blochwitz C, Nechi A, Berekovic M (2021). A comparative survey of open-source application-class RISC-V processor implementations.

[ref-13] Gaitan VG, Gaitan NC, Ungurean I (2015). CPU architecture based on a hardware scheduler and independent pipeline registers. IEEE Transactions on Very Large Scale Integration (VLSI) Systems.

[ref-14] Gary S, Stone S, Garcia S, Wray K, Rowe W, Pfau K, Liu R, Holden J, Angeles A, Willits B, Robinson K, Pérez-Celis A, Wirthlin M (2017). Dynamic SEE testing of selected architectural features of Xilinx 28 nm Virtex-7 FPGAs.

[ref-15] Ghavidel A, Sedaghat Y, Naghibzadeh M (2020). Hybrid scheduling to enhance reliability of real-time tasks running on reconfigurable devices. The Journal of Supercomputing.

[ref-16] Gruin A, Carle T, Cassé H, Rochange C (2021a). Speculative execution and timing predictability in an open source RISC-V core.

[ref-17] Gruin A, Carle T, Cassé H, Rochange C (2021b). Speculative execution and timing predictability in an open source RISC-V core.

[ref-18] Găitan VG, Zagan I (2021). An overview of the nMPRA and nHSE microarchitectures for real-time applications. Sensors.

[ref-19] Gupta N, Mandal SK, Malave J, Mandal şi R, Mahapatra N (2010). A hardware scheduler for real time multiprocessor system on chip.

[ref-20] Krishnakumar A, Arda S, Goksoy A, Mandal S, Ogras U, Sartor A, Marculescu R (2020). Runtime task scheduling using imitation learning for heterogeneous many-core systems. IEEE Transactions on Computer-Aided Design of Integrated Circuits and Systems.

[ref-21] Labrosse JJ (2002). µC/OS-II the real time kernel.

[ref-22] Leng C, Qiao Y, Hu XS, Wang H (2020). Co-scheduling aperiodic real-time tasks with end-to-end firm and soft deadlines in two-stage systems. Real-Time Systems.

[ref-23] Li J, Zhang S, Bao C (2022). DuckCore: a fault-tolerant processor core architecture Based on the RISC-V ISA. Electronics.

[ref-24] Li S, Tao Y, Tang E, Xie T, Chen R (2022). A survey of field programmable gate array (FPGA)-based graph convolutional neural network accelerators: challenges and opportunities. PeerJ Computer Science.

[ref-25] Meakin B (2010). Multicore system design with Xum: the extensible Utah multicore project. Master’s thesis.

[ref-26] Mittal S (2019). A survey on applications and architectural-optimizations of Micron’s automata processor. Journal of Systems Architecture.

[ref-27] Nacul F, Regazzoni şi M, Lajolo (2007). Hardware scheduling support in SMP architectures, design, automation & test in Europe conference & exhibition.

[ref-28] Nordström S, Lindh L, Johansson şi T, Skoglund L (2005). Application specific real-time microkernel in hardware.

[ref-29] Patterson DA, Hennessy JL (2011). Computer organization and design. Revised fourth edition: the hardware-software interface.

[ref-30] Pei S, Kim M-S, Gaudiot J-L (2016a). Extending Amdahl’s law for heterogeneous multicore processor with consideration of the overhead of data preparation. IEEE Embedded Systems Letters.

[ref-31] Pei S, Kim M-S, Gaudiot J-L (2016b). Extending Amdahl’s law for heterogeneous multicore processor with consideration of the overhead of data preparation. IEEE Embedded Systems Letters.

[ref-32] Platzer MD, Puschner P (2021). Vicuna: a timing-predictable RISC-V vector coprocessor for scalable parallel computation.

[ref-33] Pujari RK, Wild T, Herkersdorf A (2015). A hardware-based multi-objective thread mapper for tiled manycore architectures.

[ref-34] Renesas (2018). Issues with real time performance in conventional RTOS and performance improvements through HW-RTOS, HW-RTOS, real time OS in hardware, R70WP0002EJ0100.

[ref-35] Sarjoughian H, Chen Y, Burger K (2008). A component-based visual simulator for MIPS32 processors.

[ref-36] Tsai C-J, Lee Y-D (2022). Embedded TCP/IP controller for a RISC-V SoC.

[ref-37] Włostowski T, Serrano J, Vaga F (2015). Developing distributed hard-real time software systems using FPGAs and soft cores.

[ref-38] Yiu J (2019). System-on-chip design with arm Cortex-M processors. Arm education media.

[ref-39] Zagan I, Găitan VG (2022). Designing a custom CPU architecture based on hardware RTOS and dynamic preemptive scheduler. Mathematics.

